# In vivo analysis of Nef’s role in HIV-1 replication, systemic T cell activation and CD4^+^ T cell loss

**DOI:** 10.1186/s12977-015-0187-z

**Published:** 2015-07-14

**Authors:** Richard L Watkins, John L Foster, J Victor Garcia

**Affiliations:** Division of Infectious Diseases, UNC Center for AIDS Research, Genetic Medicine, University of North Carolina, Campus Box 7042, Chapel Hill, NC 27599-7042 USA

**Keywords:** HIV-1, Nef, Replication, Pathogenesis

## Abstract

**Background:**

Nef is a multifunctional HIV-1 protein critical for progression to AIDS. Humans infected with *nef*(−) HIV-1 have greatly delayed or no disease consequences. We have contrasted *nef*(−) and *nef*(+) infection of BLT humanized mice to better characterize Nef’s pathogenic effects.

**Results:**

Mice were inoculated with CCR5-tropic HIV-1_JRCSF_ (JRCSF) or JRCSF with an irreversibly inactivated *nef* (JRCSFNef*dd*). In peripheral blood (PB), JRCSF exhibited high levels of viral RNA (peak viral loads of 4.71 × 10^6^ ± 1.23 × 10^6^ copies/ml) and a progressive, 75% loss of CD4^+^ T cells over 17 weeks. Similar losses were observed in CD4^+^ T cells from bone marrow, spleen, lymph node, lung and liver but thymocytes were not significantly decreased. JRCSFNef*dd* also had high peak viral loads (2.31 × 10^6^ ± 1.67 × 10^6^) but induced no loss of PB CD4^+^ T cells. In organs, JRCSFNef*dd* produced small, but significant, reductions in CD4^+^ T cell levels and did not affect the level of thymocytes. Uninfected mice have low levels of HLA-DR^+^CD38^+^CD8^+^ T cells in blood (1–2%). Six weeks post inoculation, JRCSF infection resulted in significantly elevated levels of activated CD8^+^ T cells (6.37 ± 1.07%). T cell activation coincided with PB CD4^+^ T cell loss which suggests a common Nef-dependent mechanism. At 12 weeks, in JRCSF infected animals PB T cell activation sharply increased to 19.7 ± 2.9% then subsided to 5.4 ± 1.4% at 14 weeks. HLA-DR^+^CD38^+^CD8^+^ T cell levels in JRCSFNef*dd* infected mice did not rise above 1–2% despite sustained high levels of viremia. Interestingly, we also noted that in mice engrafted with human tissue expressing a putative protective HLA-B allele (B42:01), JRCSFNef*dd* exhibited a substantial (200-fold) reduced viral load compared to JRCSF.

**Conclusions:**

Nef expression was necessary for both systemic T cell activation and substantial CD4^+^ T cell loss from blood and tissues. JRCSFNef*dd* infection did not activate CD8^+^ T cells or reduce the level of CD4^+^ T cells in blood but did result in a small Nef-independent decrease in CD4^+^ T cells in organs. These observations strongly support the conclusion that viral pathogenicity is mostly driven by Nef. We also observed for the first time substantial host-specific suppression of HIV-1 replication in a small animal infection model.

**Electronic supplementary material:**

The online version of this article (doi:10.1186/s12977-015-0187-z) contains supplementary material, which is available to authorized users.

## Background

In individuals infected with *nef*-defective HIV-1, viral replication and pathogenesis were strongly attenuated [1–4]. Nef is a multifunctional protein and considerable effort has been made to understand which Nef activities are important for its contribution to AIDS [5–9]. These include the killing of bystander cells, maintenance of chronic viral replication leading to systemic immune system activation and blunting the host immune response [2, 10–16]. Ex vivo and in vivo models of HIV-1 infection have resulted in important advances defining Nef’s critical role for high levels of viral replication and for CD4^+^ T cell and thymocyte killing. Infection models include PBMCs, human fetal thymus organ culture, SCID-hu Thy/Liv mice and human aggregate lymphoid tissue explant from tonsil [17–26]. Unfortunately, these models could not address the systemic effects of Nef.

The bone marrow/liver/thymus (BLT) humanized mouse model has recently been employed to investigate systemic effects of HIV-1 infection. In particular, BLT humanized mice have been inoculated with HIV-1_JRCSF_ (JRCSF) that has the CCR5 tropism predominantly found in infected individuals [27]. Denton et al. found high levels of replication by JRSCF and a significant cytopathic effect on CD4^+^CCR5^+^ T cells. Nie et al. [28] also found high levels of viral replication and targeted killing of CD4^+^CCR5^+^ T cells in NOG-hCD34 mice. Finally, Dudek et al. [29] also reported high levels of JRCSF replication in NOD/SCID BLT and NOD/SCID/IL2Rγc^−/−^ BLT mice. However, the role of Nef in HIV replication and CD4^+^ T cell depletion in the context of a CCR5-tropic virus has not been reported. In addition, Long et al. [30] infected BLT mice with JRCSF and observed systemic activation of peripheral blood CD8^+^ T cells but the role of Nef was not investigated. Therefore, we have extended previous studies to compare JRCSF infection with infection by JRCSF modified to contain an irreversibly inactivated *nef* (JRCSFNef*dd*). In BLT humanized mice, Nef was found to have a limited role in JRCSF replication, but was necessary for systemic T cell activation and CD4^+^ T cell loss in peripheral blood and in tissues. This was the case for multiple BLT mouse human tissue cohorts. However, in one exceptional cohort expressing an HIV-1 protective HLA-B allele (B42:01), the absence of Nef expression led to a 200-fold reduction in viral loads. This reduction was not observed in mice infected with the wildtype virus expressing Nef. This is the first demonstration of a host specific effect on viral load in an HIV-1 infection model.

## Results

### Infection of BLT humanized mice with JRCSF and JRCSFNef*dd*

**Figure 1:**
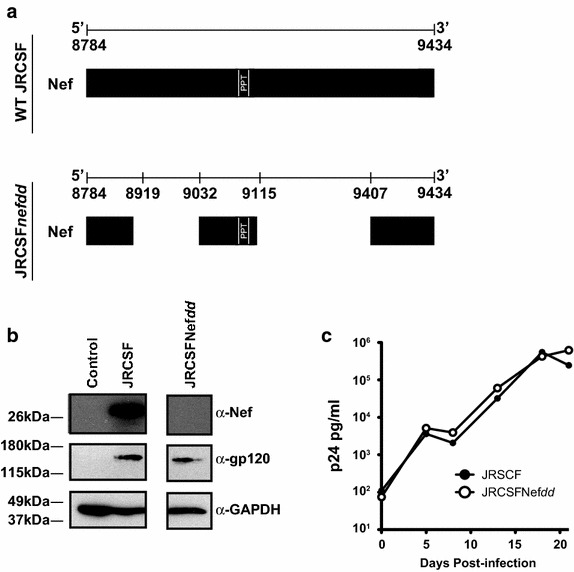
HIV-1_JRCSF_ with a truncated *nef*. **a** *Upper panel* schematic representation of wild type JRCSF *nef* (WT JRCSF) is presented. Nucleotides 8784–9434 in NCBI accession number, M38429, represent the *nef* coding sequence. *PPT* polypurine tract. *Lower* *panel*, a schematic presentation of *nef* with two deletions (JRCSFNef*dd*). A 114 bp deletion 5′ of the PPT from 8919 to 9032 inclusive was introduced into *nef*. The downstream sequence was shifted to the +1 reading frame by cutting with *Xho*I and filling in with Klenow (“Methods”). The 293 bp deletion 3′ of the PPT shifted downstream *nef* sequence to reading frame to +2. **b** The proviral clones for JRCSF and JRCSFNef*dd* were transfected into 293T cells and after 2 days Nef and Env expressions assessed by Western blots, GADPH is a loading control. **c** Replication competence of JRCSFNefdd was not diminished by loss of Nef as determined by p24^*gag*^ production in CEM cells expressing CCR5. Cells were infected at 1 × 10^5^ TCIU at an MOI 0.01 and the production of p24^*gag*^ was followed for 21 days.

We compared the infection of humanized BLT mice with the CCR5-tropic JRCSF and JRCSF with an irreversibly inactivated *nef* (JRCSFNef*dd*) to discern the phenotypic differences between wild type and *nef*(−) virus (Figure 1a). The deletions were made to reflect the truncations of *nef* found in patients reported to have been infected with a *nef*(−) virus [1, 3, 31, 32]. Though the proviral clone for JRCSFNef*dd* did not express Nef it did produce wild type levels of Env (Figure 1b). Further, in Figure 1c we observed that the *nef* deletions did not affect viral replication of this virus [33].

**Figure 2:**
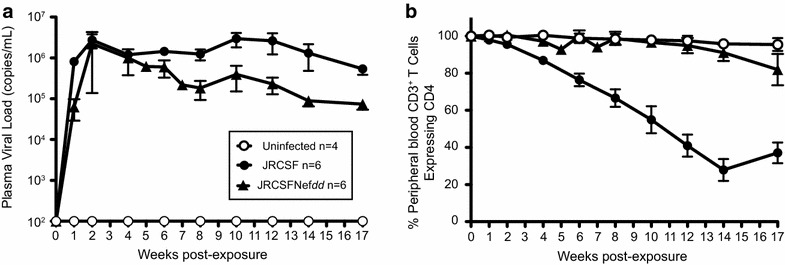
Viral load analysis and PB CD4^+^ T cell loss in mice infected with JRCSF and JRCSFNef*dd.* **a** Viral loads (copies of viral RNA per ml of plasma) were plotted for BLT mice that were exposed to 90,000 TCIU. JRCSF, JRCSFNef*dd* and uninfected mice were followed for 17 weeks. **b** The percent of CD4^+^ T cells out of total T cells in peripheral blood are plotted for the three groups of mice in **a**.

In Figure 2a, the levels of virus in blood following intravenous injection of JRCSF or JRCSFNef*dd* [9 × 10^4^ tissue culture infectious units (TCIU)] were monitored for 17 weeks. Both viruses showed rapid increases of viral RNA in blood with high levels of virus throughout the course of infection. Peak viral loads for the two viruses were not significantly different (JRCSF, 4.71 × 10^6^ ± 1.23 × 10^6^ copies of viral RNA per ml versus JRCSFNef*dd*, 2.31 × 10^6^ ± 1.67 × 10^6^). However, at 8 weeks the average viral load for JRCSFNef*dd* mice was lower than the average viral load for JRCSF mice (0.18 × 10^6^ ± 0.09 × 10^6^ and 1.24 × 10^6^ ± 0.37 × 10^6^, respectively; p < 0.033) but this significant difference was not observed at later time points because JRCSFNef*dd* viral loads displayed considerable variation over time (Additional file 1: Figure S1).

We also monitored CD4^+^ T cells in blood post JRCSF inoculation over the course of infection. Our results show a slow, 17 week decline in CD4^+^ T cells while CD4^+^ T cell levels in uninfected mice remained unchanged (Figure 2b). These slow losses in CD4^+^ T cells are in contrast with those previously reported with X4-tropic HIV-1_LAI_ (LAI) that rapidly depleted CD4^+^ T cells from blood following inoculation [32]. Conversely, JRCSFNef*dd* infected BLT mice showed no reduction in peripheral blood CD4^+^ T cells (Figure 2b) which is similar to what was previously observed during the course of LAINef*dd* infection under similar experimental conditions [32].

### CD4^+^ T cell levels in tissues of mice infected with JRCSFNef*dd* are higher than those in BLT mice infected with JRCSF

**Figure 3:**
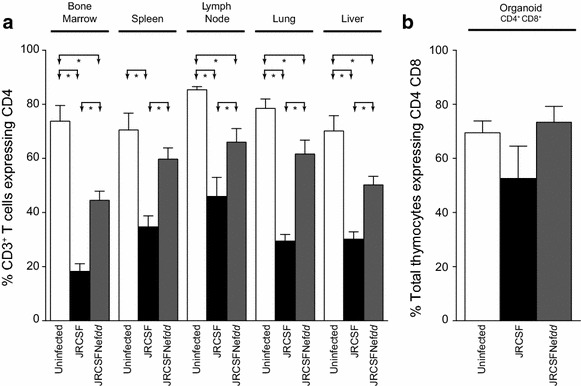
Systemic CD4^+^ T cell but not thymocyte loss in mice infected with JRCSF and JRCSFNef*dd*. **a** CD4^+^ T cell analysis was performed on five organs from un-exposed BLT mice (uninfected, n = 4), JRCSF infected BLT mice (n = 5) and JRCSFNef*dd* infected BLT mice (n = 6). Significant differences are indicated by *lines* and *arrows* above respective bars (*p < 0.05). **b** The same analysis as in **a** is presented for CD4^+^CD8^+^ double positive thymocytes relative to total thymocytes. No statistical differences were observed. *Error bars* are the mean ± SEM.

The BLT mice from Figure 2 were sacrificed and CD4^+^ T cells present in bone marrow, spleen, lymph node, lung and liver were analyzed by flow cytometry (Figure 3a). In JRCSF infected mice, all five organs exhibited significant drops in the levels of CD4^+^ T cells. In four of five organs, the JRCSFNef*dd* infected mice also had reduced levels of CD4^+^ T cells, the exception being spleen. However, the loss of CD4^+^ T cells as a result of JRCSFNef*dd* infection was not as great as for JRCSF (p < 0.05 for bone marrow, spleen, lymph node, lung and liver, Figure 3a). In the case of CD4^+^CD8^+^ thymocytes, there was no significant reduction noted for JRCSF or JRCSFNef*dd* (Figure 3b). These results show that the CD4^+^ T cell depletion observed during the course of JRCSF’s infection is blunted in the absence of Nef expression.

### Analysis of systemic T cell activation during the course of infection with JRCSF and JRCSFNef*dd*

**Figure 4:**
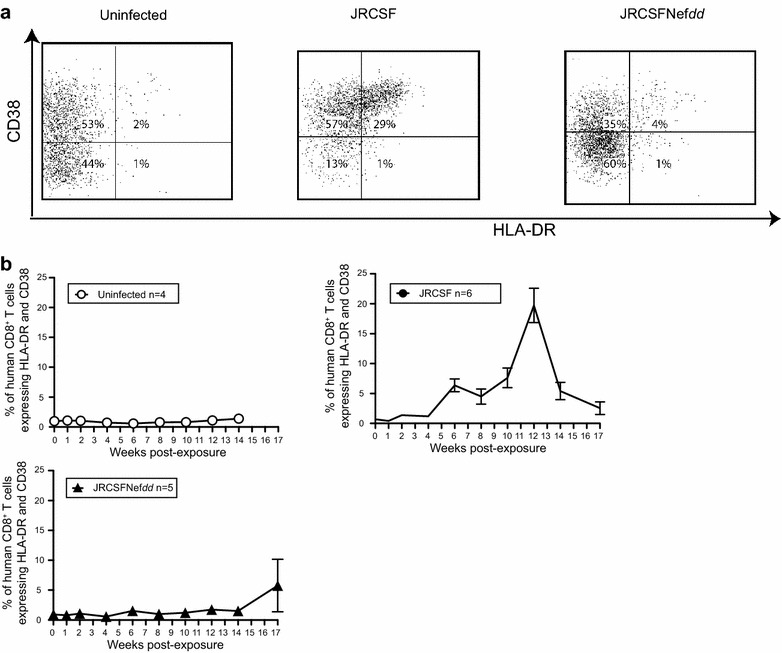
Systemic T cell activation during infection with JRCSF and JRCSFNef*dd*. Systemic T cell activation was assessed by the levels of HLA-DR^+^CD38^+^CD8^+^ relative to total CD8^+^ T cells in blood. **a** Representative *dot plots* for the presence of activated CD8^+^ T cells in blood of uninfected, JRCSF-infected (JRCSF 1) and JRCSFNef*dd*-infected (JRCSFNef*dd* 6) mice. Gating protocol was performed as indicated in “Methods”. Data shown are from 12 wpi. **b** Aggregate data of CD8^+^ T cell activation over the course of 17 weeks of infection is shown for uninfected (n = 4), JRCSF infected (n = 6) and JRCSFNef*dd* infected (n = 5) mice.

JRCSF infected mice display a relatively slow decline in peripheral blood (PB) CD4^+^ T cells but JRCSFNef*dd* infected mice did not lose these cells (Figure 2b). The possibility of an association of CD4^+^ T cell loss with systemic T cell activation was investigated [30]. Representative flow cytometric analyses of HLA-DR^+^CD38^+^CD8^+^ T cells in blood at 12 weeks are presented in Figure 4a. Levels of activated CD8^+^ T cells were quite low in uninfected mice but greatly increased during JRCSF infection. In contrast, JRCSFNef*dd* infection had little effect on T cell activation despite peak viral loads that were not significantly lower than JRCSF (Figure 2a). In Figure 4b, the aggregate time courses for HLA-DR^+^CD38^+^CD8^+^ T cells in blood are shown with individual plots presented in Additional file 2: Figure S2. Uninfected mice did not have elevated levels of activated CD8^+^ T cells at any point during the experiment and JRCSFNef*dd* infected mice had nearly identical results with the exception of a single mouse (JRCSFNef*dd* 6) at a single time point (week 17) during the entire course of the study (Additional file 2: Figure S2). Interestingly, for JRCSF infected mice both the activation of CD8^+^ T cells (JRCSF 6.3 ± 1.1% vs uninfected 1.7 ± 0.5%, p = 0.0095, p = 0.0008; Figure 4b) and the loss of PB CD4^+^ T cells (JRCSF 6.4 ± 1.1% vs uninfected mice 0.6 ± 0.1%; p = 0.0095; Figure 2b) were first clearly evident at 6 weeks. T cell activation remained elevated and PB CD4^+^ T cell continued to decline for 14 weeks (Figures 2b, 4b). Therefore, the appearance of activated CD8^+^ T cells and loss of CD4^+^ T cells in blood were tightly correlated. At 12 weeks, further activation occurred (JRCSF, 19.7 ± 2.9% HLA-DR^+^CD38^+^CD8^+^ T cells versus uninfected, 1.1 ± 0.3%, p = 0.0003 and versus JRCSFNef*dd*, 1.7 ± 0.5% p = 0.0008). There was no dramatic effect of this spike on viral load or the steady decline in PB CD4^+^ T cells (Figure 2a, b).

**Figure 5:**
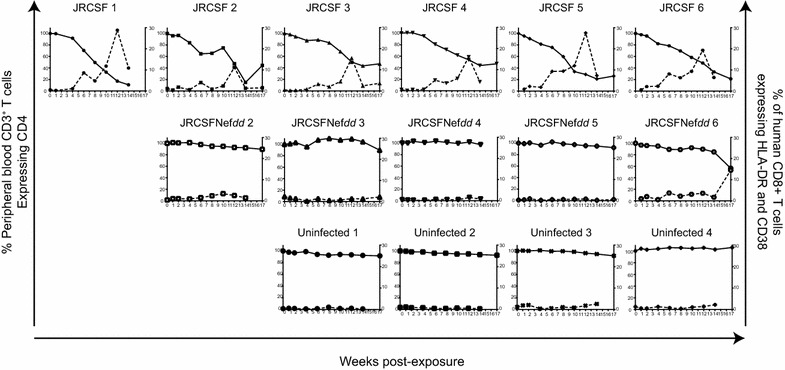
Systemic T cell activation is compared to PB CD4^+^ T cell loss in individual mice. Plots for individual mice of CD4^+^ T cells (*left y axis*) and of HLA-DR^+^CD38^+^CD8^+^ T cells (*right y axis*) over the infection time course are shown. *Top* *row* all six JRCSF infected mice show increases in CD8^+^ T cell activation concomitant to PB CD4^+^ T cell loss. *Middle* *row* JRCSFNef*dd* infected BLT mice show very low T cell activation and no CD4^+^ T cell loss except for JRCSFNef*dd* 6 which had a drop in CD4^+^ at week 17. *Bottom* *row* uninfected mice show very low T cell activation and no CD4^+^ T cell loss.

Time courses of T cell activation for the individual control or JRCSF and JRCSFNef*dd* infected mice are presented in Figure 5. In each JRCSF infected mouse, the early appearance of CD8^+^ T cell activation is temporally associated with the initial loss of CD4^+^ T cells in blood. Conversely, there was no elevation of HLA-DR^+^CD38^+^CD8^+^ T cells and no loss of CD4^+^ T cells in PB for either uninfected or JRCSFNef*dd*-infected BLT mice except as noted above for JRCSFNef*dd* 6 at 17 weeks. At this singular, late time point, the association between T cell activation and CD4^+^ T cell decline is maintained despite the absence of Nef expression (Additional file 2: Figure S2). In sum, our results demonstrate a strong and highly consistent linkage of CD8^+^ T cell activation to the loss of PB CD4^+^ T cells in JRCSF infected mice.

### Specific suppressive effect on JRCSFNef*dd* viral load in mice reconstituted with cells and tissue expressing HLA B42:01

**Figure 6:**
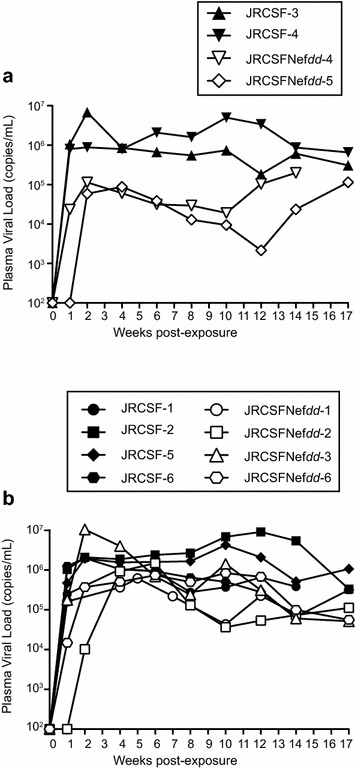
Viral load time courses for JRCSFNef*dd* infected mice and JRCSF infected mice with Cohort 1 tissue. **a** The data in Figure 2a is re-plotted to separate the four Cohort 1 mice from the other eight mice. Two Cohort 1 mice were infected with JRCSF and two were infected with JRCSFNef*dd*. **b** The remaining eight mice were plotted. These mice were from Cohorts 2–5; see Table 1).

Dudek et al. investigated infection with JRCSF of eight BLT humanized mice tissue cohorts with many HLA haplotypes represented. The only cohort-specific reduction in viral load found was with mice implanted with B57-expressing human tissue. Mice with human cells expressing this protective haplotype exhibited a four-fold reduction in viral load relative to all other haplotypes investigated including the protective allele, B27 [29]. We did not have tissue with the B57 haplotype and consistent with Dudek et al. we observed no cohort-specific suppression of viral load with JRCSF infection. Nor did we observe reductions in viral loads with JRCSFNef*dd* infections except for one cohort of mice designated Cohort 1 (Table 1). Cohort 1 has HLA haplotypes, A23:01, A30:01, B42:01, B53:01, C08:01, C17:01. In a report based on a large population of South African HIV-1 positive individuals, B42:01 was one of the few HLA-B haplotypes found to have significantly lower viral loads [34]. Two Cohort 1 mice were infected with JRCSFNef*dd* and had consistently lower viral loads than two Cohort 1 JRCSF mice at every time point (Figure 6a). At week 10, this reduction reached 200-fold. Comparisons of the four JRCSF and four JRCSFNef*dd* mice from Cohorts 2, 3, 4, and 5 gave considerable overlap in the viral loads with the JRCSF and JRCSFNef*dd* infected mice (Figure 6b). On the basis of these results, we hypothesize that JRCSFNef*dd* infected mice can have substantially reduced viral burdens relative to JRCSF mice but the reduction is dependent on the genotype of the engrafted tissue.

**Table 1:** Haplotype of tissues/CD34 stem cells used for the construction of the BLT humanized mice used

Cohort 1	A23:01 A30:01 B42:01 B53:01 C08:01 C17:01	JRCSF 3, 4; JRCSFNef*dd* 4, 5
Cohort 2	A02:01 A02:01 B35:17 B52:01 C03:03 C04:04	JRCSF 2; JRCSFNef*dd* 3
Cohort 3	A02:01 A03:01 B35:01 B44:02 C04:01 C05:01	JRCSF 1
Cohort 4	A11:01 A24:02 B35:01 B35:24 C04:01 C04:01	JRCSF 5, 6; JRCSFNef*dd* 6
Cohort 5	*A02:06* A34:01 B39:05 B40:02 C07:02 C15:02	JRCSFNef*dd* 2
Cohort 6	ND	JRCSFNef*dd* 1

Multiple BLT mice constructed from the same human tissue are designated by a cohort number. The lower level of PB JRCSFNef*dd* viral RNA compared to JRCSF viral RNA in Cohort 1, but none of the others, led us to have the MHCI haplotypes determined. A02:06 in Cohort 5 is in italics because it appears to be a previously undocumented A2 variant (similar to A*02:06). There is a nucleotide substitution in exon 3 that is not present in the HLA database. As it is located in exon 3 and encodes a non-synonymous substitution (Histidine > Leucine at codon 114), it may impact peptide binding.

## Disscusion

We have extensively characterized infection of BLT humanized mice with a wild type R5-tropic virus (JRCSF) and an isogenic *nef*(−) virus (JRCSFNef*dd*). Following JRCSF infection, there is a progressive 17 week decline in CD4^+^ T cells in blood to about 25% of the levels found in uninfected mice. Also at 17 weeks, CD4^+^ T cells in organs were reduced to 20–40% of the levels in uninfected mice. These substantial pathogenic effects are, nonetheless, less aggressive than the CD4^+^ T cell losses previously reported for the CXCR4-tropic HIV-1 LAI in this same model and under similar experimental conditions [32]. However, one stark difference between JRCSF and LAI infection was that thymocytes were present at near normal levels throughout JRCSF infection but massively depleted by LAI. The maintenance of thymocyte viability despite R5-tropic infection may reflect the paucity of CCR5 expressing cells in thymocytes [27]. Interestingly, Jamieson et al. [24] reported severe losses of thymic organoid cells with X4-tropic NL-43 infection of SCID–hu mice but little evidence of pathogenicty for R5-tropic JRCSF. Not surprisingly, our results reflect these earlier results with regard to thymocytes. However, previously unreported is the loss of about 75% of CD4^+^ T cells from blood and tissues. In contrast to the rapid loss of CD4^+^ T cells in BLT mice with LAI infection, a largely intact human thymic organ during JRCSF infection could replenish CD4^+^ T cells in the periphery and slow the net loss of CD4^+^ T cells. The loss of thymocytes during X4-tropic LAI infection would not allow buffering the CD4^+^ T cell levels in tissues resulting in dramatic depletion of these cells [32]. These considerations suggest that R5-tropic infection may be inherently as cytotoxic as X4-tropic infection but exhibits less drastic effects because of different cellular targets.

Another difference between LAI and JRCSF infection was that peak viral loads were reduced about sevenfold with LAINef*dd* infection compared to wild type but a only a twofold reduction in peak viral load was noted with JRCSFNef*dd* (Figure 2), [32]. HIV-1 _JRFL_ (JRFL) is closely related to JRCSF and Usami and Gottlinger reported that JRFL and *nef*(−) JRFL have similar infectivities. The B-C hairpin of the V2 region fails to respond to Nef and prevents the functioning of Nef to enhance virion infectivity [35]. JRCSF has 90% identical residues in the B-C hairpin to JRFL which may account for the low impact of the loss of Nef expression on JRCSFNef*dd* peak viral load. However, arguing against this explanation for the small impact of *nef* inactivation on viral replication is that JRCSF replicated to high viral loads with or without *nef*.

The T cell activation data (Figures 4, 5) in combination with the T cell loss in tissues data (Figure 3a) for JRCSF and JRCSFNef*dd* gives evidence for two mechanisms of JRCSF cell toxicity. One mechanism observed with JRCSFNef*dd* infection is Nef and systemic T cell activation independent. It results in a relatively low level of killing and is only found in tissues as CD4^+^ T cells in blood are not reduced. The second mechanism is Nef-dependent and may account for the parallel loss of CD4^+^ T cells in blood and increased PB CD8^+^ T cell activation. The higher level of killing in tissues by JRCSF is also likely to be the result of Nef expression and/or T cell activation (Figure 3a). Killing of CD4^+^ T cells has been linked to systemic T cell activation [30] and we found a strong association of PB CD4^+^ T cell loss with systemic T cell activation. Since PB CD4^+^ T cells are not productively infected by HIV-1 the mechanism of PB CD4^+^ T cell loss caused by JRCSF infection is likely to be indirect. Elevated CD8^+^ T cell activation was first noted at 6 weeks when PB CD4^+^ T cell have clearly begun to decline and continued to increase to very high levels by 12 weeks (Figure 2b, 4b). The expression of Nef was necessary for these effects as JRCSFNef*dd* failed to cause activation or CD4^+^ T cell loss.

The percent of CD4^+^ T cells that express CCR5 is relatively high in bone marrow, lung and liver and these cells are lost with JRCSF infection [27]. It is not known if the cytotoxic effect of JRCSF in these organs is indirect through systemic T cell activation or direct as a result of one or more of Nef’s numerous activities. Direct Nef effects have been proposed for killing bystander cells by induction of apoptosis [14, 15, 36–39]. However, we also observed reduced but significant losses of CD4^+^ T cells in tissues with JRCSFNef*dd* infection. To explain the loss of CD4^+^ T cells in tissues following JRCSFNef*dd* infection, mechanisms that are independent of Nef and systemic T cell activation are required. One possibility involves pyroptotic death of abortively infected cells [40, 41]. In JRCSFNef*dd*-infected mice, the combined effects of having high peak viral loads in the absence of systemic T cell activation may be expected to favor abortive infection. An alternate mechanism could be the direct binding to bystander cells by Env [42, 43]. The full elucidation of the mechanism of Nef- and activation-independent CD4^+^ T cell loss will be important for complete understanding of CD4^+^ T cell loss in R5-tropic HIV-1 infection. The ability of Nef to modulate cellular protein kinases may be critical in this regard [5, 38, 44, 45].

In general, we did not observe significant reductions in viral loads for JRCSFNef*dd* infected mice compared to JRCSF infected mice but there was a reduction of JRCSFNef*dd* viral load in BLT mice from one of six cohorts. Specifically, there was a 200-fold reduction of viral load in JRCSFNef*dd* infected mice compared to JRCSF infected mice sharing Cohort 1 reconstitution. The host factor responsible for the reduction in viral load has not been identified, however, B42:01 is one of the HLA haplotypes for Cohort 1 and B42:01 has a negative impact on viral loads of HIV-1 positive individuals [34]. The reduction in viral loads observed for the JRCSFNef*dd* infected Cohort 1 mice suggest the intriguing possibility that a weak anti-HIV effect by B42:01 in BLT mice is greatly enhanced when Nef is absent. The converse conclusion is that the all other haplotypes present in this study are ineffective in reducing viral load even with *nef*(−) virus. In future studies, it will be important to screen HLA haplotypes to determine the anti-viral effects of multiple HLA haplotypes, especially the well-known protective allele B57. In this regard, the Sydney Blood Bank Cohort of patients infected with *nef*(−) HIV-1 all had greatly delayed disease progression, however of special interest is patient C135 with the B57 haplotype that was negative for virus in blood for 29 years [46, 47].

## Conclusions

We have demonstrated that in the context of the CCR5-tropic HIV-1 infection the accessory protein Nef is required for peripheral blood CD4^+^ T cell depletion. In addition, we observed an association between peripheral blood CD8^+^ T cell activation and the loss of CD4^+^ T cells. Neither activation nor CD4^+^ T cell loss was observed in mice infected with JRCSFNef*dd*. The requirement of Nef expression for CD8^+^ T cell activation during of HIV-1 infection suggests that Nef plays a critical role in the widespread nature of HIV-1 cytotoxicity. Moreover, this Nef-dependent activation is linked to the loss of CD4^+^ T cells but the mechanism is not known. A relatively small, Nef-independent cytotoxic T cell effect was also observed. This loss of CD4^+^ T cells was restricted to tissues. The significance of this finding is unknown but overall pathogenicity appears to be largely driven by Nef. Future investigations will pursue understanding the long-elusive mechanism behind the fundamental phenomenon of CD4^+^ T cell loss during HIV-1 infection.

We also observed a reduced ability of JRCSFNef*dd* to replicate in mice from a specific cohort of identical engrafted human tissue. Of great interest, this cohort expressed B42:01 which is an allele significantly associated with reduced viral burdens by studies of large populations of HIV-1 infected individuals. Thus, the BLT humanized mouse infected with JRCSFNef*dd* may provide a platform to independently identify protective HLA alleles.

## Methods

### Preparation of humanized BLT mice

Humanized BLT mice were prepared as previously described [27, 29, 30, 32, 48–55]. Briefly, thymus/liver implanted NOD/SCID IL-2γ^−/−^ mice (The Jackson Laboratories, Bar Harbor, ME, USA) were transplanted with autologous human CD34^+^ cells isolated from fetal liver (Advanced Bioscience Resources, Alameda, CA, USA). Reconstituted mice have a highly representative human immune system. Multiple mice reconstituted from a single source of autologous thymus/liver implant and human CD34^+^ cells represent a single cohort. Mice from seven cohorts were used (Table 1). Human reconstitution in the peripheral blood of these mice was monitored periodically by flow cytometry prior to use (FACSCanto; BD Biosciences). Mice were maintained at the Division of Laboratory Animal Medicine, University of North Carolina at Chapel Hill (UNC-CH) in accordance with protocols approved by the UNC-CH Institutional Animal Care and Use Committee.

### Cell lines and culture conditions

293T and TZM-bl cells were maintained in Dulbecco’s modified Eagle’s medium (DMEM; Cellgro, Herndon, VA, USA) supplemented with 10% fetal bovine serum (FBS; Cellgro), 100 IU/ml of penicillin, 100 μg/ml streptomycin, and 2 mM glutamine (Cellgro) in 10% CO_2_ at 37°C.

### Proviral clones

The proviral clone, pYK-JRCSF (accession #M38429), was described by Koyanagi et al. [56]. pYK-JRCSFNef*dd* was constructed by first creating a 5′ deletion upstream of the PPT. The *Xho*I/*Acc*65I fragment was removed and blunt ends were made with Klenow followed by religation. The reconstituted *Xho*I site was cut and treated with Klenow and religated. In the 3′ half of *nef* 288 bases were deleted by site directed mutagenesis as previously described for pLAInef*dd* [32].

### Exposure of BLT humanized mice to JRCSF and JRCSFNef*dd*, assay of viral production, tissue harvesting and cytometric analyses

Stocks of JRCSF and JRCSFNef*dd* were prepared as previously described [44, 57]. Briefly, proviral clones were transfected into 293T cells. Viral supernatant was collected 48 h after transfection and diluted in Dulbecco’s modified Eagle’s medium (DMEM) supplemented with 10% fetal bovine serum, 100 IU penicillin/ml, 100 μg/ml streptomycin, and 2 mM glutamine. TZM-bl cells were infected in 12-well tissue culture plates with 0.4 ml of virus at multiple dilutions in medium for 2 h. Then, 1.0 ml of supplemented DMEM was added and the plates incubated overnight. Virus containing medium was removed the next day, replaced with fresh DMEM plus 10% fetal bovine serum and the incubation continued for 24 h. The cells were fixed and stained with 5-bromo-4-chloro-3-indolyl-β-d-galactopyranoside (40 h after first exposure to virus). Blue cells were counted directly to determine infectious particles per mL. Each titer of these viral stocks was performed in triplicate and at least two different titer determinations were performed for each batch of virus. p24^*gag*^ was determined for each virus preparation with the ELISA HIV-1 p24 antigen capture assay from Advanced Bioscience Laboratories Inc. (Cat. No. 5421).

Intravenous exposure of BLT mice with infectious virus was conducted via tail vein injection with indicated tissue culture infectious units (TCIU). Viral load in peripheral blood of infected mice was monitored longitudinally by quantitative real-time PCR using Taqman RNA to-C_T_™ 1-step kit from Applied Biosystems, USA [50, 58]. The sequences of the forward and reverse primers and the Taqman probe for PCR were: 5′-CATGTTTTCAGCATTATCAGAAGGA-3′, 5′-TGCTTGATGTCCCCCCACT-3′, and 5′-FAM CCACCCCACAAGATTTAAACACCATGCTAA-Q-3′, respectively.

CD4^+^ and CD8^+^ T cell levels were monitored by flow cytometric analysis as previously described [27, 53, 55]. Immunophenotyping was performed on blood samples collected longitudinally and on mononuclear cells isolated from tissues at harvest. Whole peripheral blood (PB) from humanized mice was analyzed according to the BD Biosciences Lyse/Wash protocol (Cat. No. 349202) as we have previously described [59]. Briefly, following antibody labeling of whole blood, red blood cells were lysed. The remaining cells were washed, fixed and the sample was analyzed by flow cytometry. Tissue mononuclear cell isolations and immunophenotyping analyses were also performed according to published methods [27, 53, 55]. Flow cytometric gating for CD4 and CD8 cell surface expression was performed as follows: (step 1) forward and side scatter properties were utilized to set a live cell gate; (step 2) live cells were then analyzed for expression of the human pan-leukocyte marker CD45; (step 3) human leukocytes were then analyzed for hCD3 and (step 4) T cells or thymocytes were analyzed for hCD4 and hCD8 expression.

The panel of antibodies for analysis of CD8^+^ T cells double positive for CD38^+^ and HLA-DR^+^ was CD8 FITC (SK1), HLA-DR, PE (TU36) or IgG2bκ PE, CD4 PerCP (SK3), CD3 PE-Cy7 (SK7), CD38 APC (HB7) or IgG1κ APC, and CD45 APC-Cy7 (2D1) (all purchased from BD Biosciences). Gating was performed as follows: (step 1) forward and side scatter properties were utilized to set a live cell gate; (step 2) live cells were then analyzed for expression of the human pan-leukocyte marker CD45; (step 3) human leukocytes were then analyzed for CD3; (step 4) T cells were analyzed for CD4 and/or CD8 expression; (step 5) activation of human CD8^+^ T cells was analyzed for HLA-DR and CD38 expression [30]. Gates defining HLA-DR and CD38 expression were set with isotype-matched fluorophore-conjugated antibodies.

### Viral replication in vitro

The human T cell line, CEM (NIH AIDS Reagent Program), was modified to express CCR5 [33]. Cells were infected with virus stocks at 1 × 10^5^ TCUI at an MOI of 0.01 in complete RPMI containing 2 µg/ml polybrene at 37°C, 5% CO_2_ for 4 h. The cells were washed extensively with PBS and cultured at 37°C, 5% CO_2_ in complete RPMI. Cell cultures were passaged at 0, 5, 8, 13, 18, and 21 days post-infection and a sample of the culture supernatant was collected for quantification of viral capsid protein by p24^*gag*^ ELISA.

### HLA haplotyping

HLA haplotypes were determined by sequence based typing using SeCore HLA typing reagents (Life Technologies) on an ABI3500 capillary sequencer. Data analysis was performed using uType software (Life Technologies). When necessary, ambiguous allele combinations were resolved with sequence specific oligonucleotide probe hybridization (ThermoFisher). DNA was extracted a Promega Maxwell automated DNA extractor and kits.

### Statistical analysis

Student t test was conducted using Prism Version 5 (Graph Pad). All data were plotted as mean ± SEM.

## Additional files

Additional file 1:
**Figure S1.** Viral loads plotted for individual mice **(A)**. The viral loads for each of the six BLT humanized mice infected with JRCSF from Figure 2A are plotted separately. **(B)** The viral loads for the six BLT humanized mice infected with JRCSFNef*dd* from Figure 2A are presented as individual plots. JRCSF infected mice are from four different cohorts and JRCSFNef*dd* infected mice are from five different cohorts. The cohorts were distributed as follows. Cohort 1—JRCSF 3, 4 and JRCSFNef*dd* 4,5; Cohort 2—JRCSF 2, and JRCSFNef*dd* 3; Cohort 3—JRCSF 1; Cohort 4—JRCSF 5, 6 and JRCSFNef*dd* 6; Cohort 5—JRCSFNef*dd* 2; Cohort 6—JRCSFNef*dd* 1. Additional file 2:
**Figure S2.** Time course of T cell activation plotted for individual mice. **(A)** Individual JRCSF infected mice are shown. **(B)** Individual JRCSFNef*dd* mice are shown.
