# Supplementary figures and images for: In vivo analysis of Nef’s role in HIV-1 replication, systemic T cell activation and CD4+ T cell loss
Source: Retrovirology. 2015 Jul 14;12:61. doi: 10.1186/s12977-015-0187-z (PMC4501112; doi:10.1186/s12977-015-0187-z)

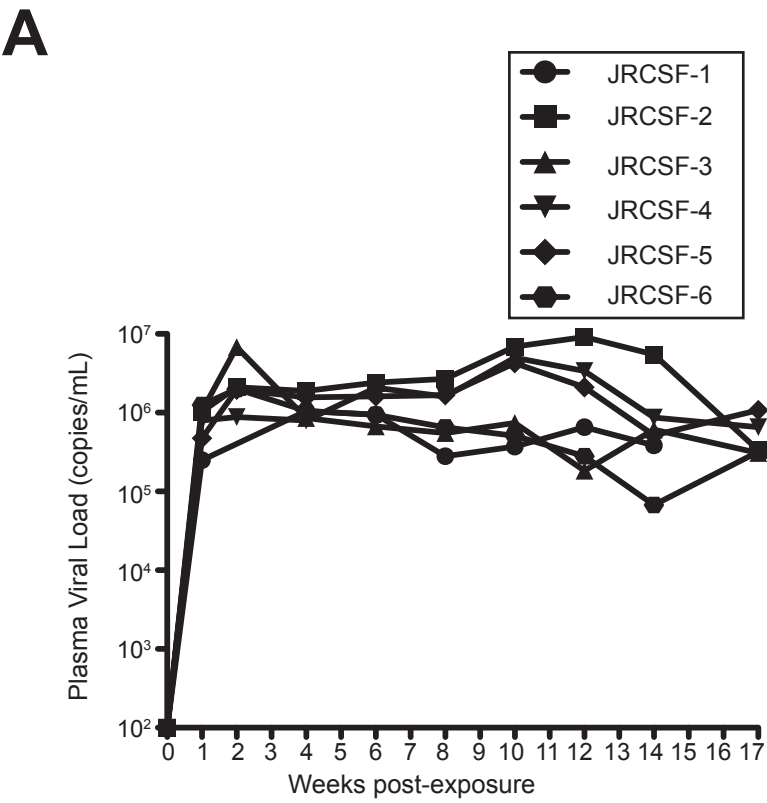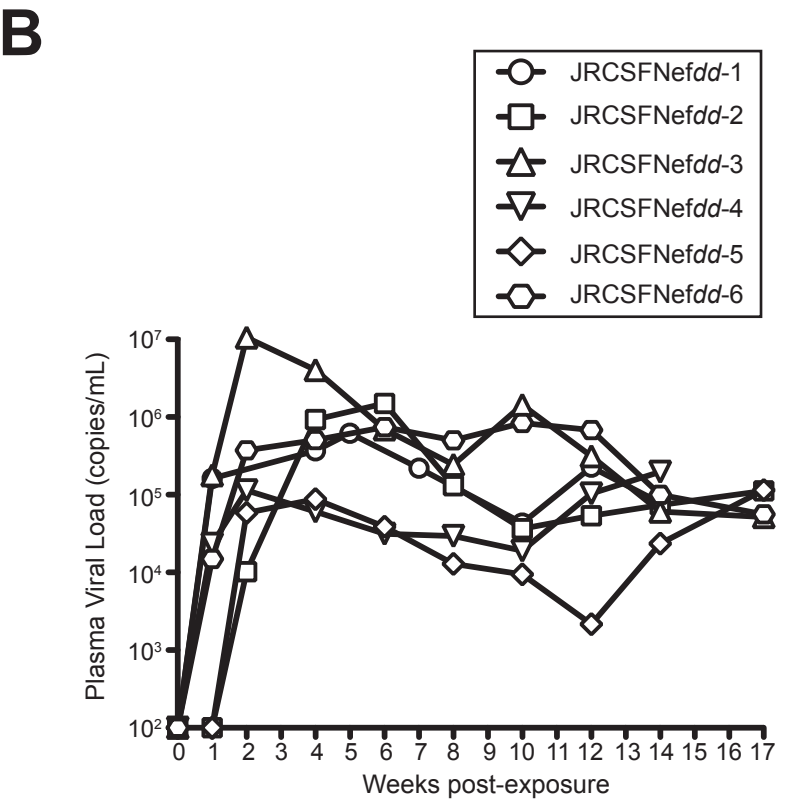

**Figure S1.**

Supplement: Additional file 1: — Figure S1. Viral loads plotted for individual mice (A). The viral loads for each of the six BLT humanized mice infected with JRCSF from Figure 2A are plotted separately. (B) The viral loads for the six BLT humanized mice infected with JRCSFNefdd from Figure 2A are presented as individual plots. JRCSF infected mice are from four different cohorts and JRCSFNefdd infected mice are from five different cohorts. The cohorts were distributed as follows. Cohort 1—JRCSF 3, 4 and JRCSFNefdd 4,5; Cohort 2—JRCSF 2, and JRCSFNefdd 3; Cohort 3—JRCSF 1; Cohort 4—JRCSF 5, 6 and JRCSFNefdd 6; Cohort 5—JRCSFNefdd 2; Cohort 6—JRCSFNefdd 1. [file 12977_2015_187_MOESM1_ESM.pdf]

A

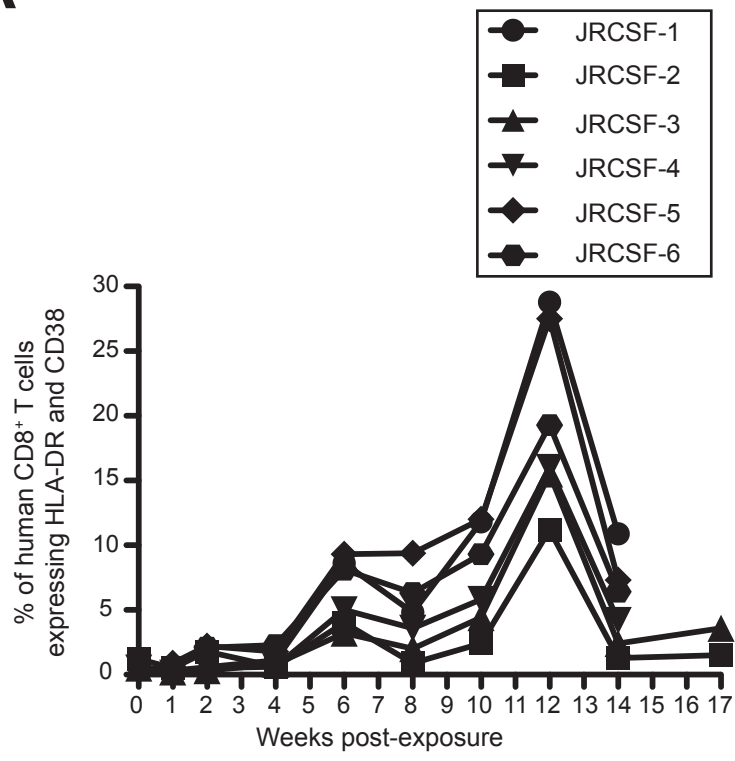

B

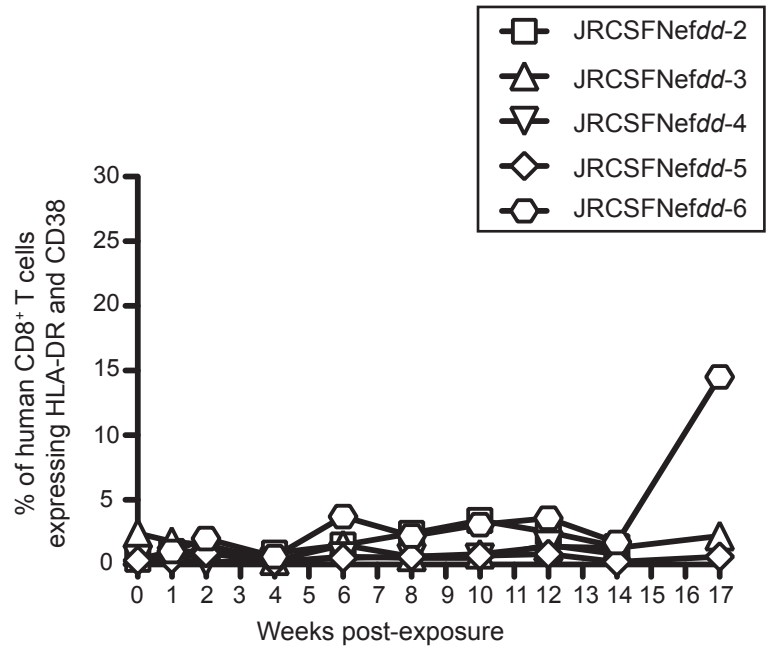

Figure S2.

Supplement: Additional file 2: — Figure S2. Time course of T cell activation plotted for individual mice. (A) Individual JRCSF infected mice are shown. (B) Individual JRCSFNefdd mice are shown. [file 12977_2015_187_MOESM2_ESM.pdf]
